# MIR221HG Is a Novel Long Noncoding RNA that Inhibits Bovine Adipocyte Differentiation

**DOI:** 10.3390/genes11010029

**Published:** 2019-12-26

**Authors:** Mingxun Li, Qisong Gao, Zhichen Tian, Xubin Lu, Yujia Sun, Zhi Chen, Huimin Zhang, Yongjiang Mao, Zhangping Yang

**Affiliations:** 1 Key Laboratory of Animal Genetics & Breeding and Molecular Design of Jiangsu Province, Yangzhou University, Yangzhou 225002, China; limingxun@yzu.edu.cn (M.L.); MZ120181011@yzu.edu.cn (Q.G.); 171902314@yzu.edu.cn (Z.T.); dx120180094@yzu.edu.cn (X.L.); zhichen@yzu.edu.cn (Z.C.); hmzhang@yzu.edu.cn (H.Z.); cattle@yzu.edu.cn (Y.M.); 2 Joint International Research Laboratory of Agriculture and Agri-Product Safety of Ministry of Education of China, Yangzhou University, Yangzhou 225002, China; ysunshine@yzu.edu.cn

**Keywords:** long noncoding RNA, MIR221HG, subcellular localization, adipocyte differentiation

## Abstract

Adipogenesis is a complicated but precisely orchestrated process mediated by a series of transcription factors. Our previous study has identified a novel long noncoding RNA (lncRNA) that was differentially expressed during bovine adipocyte differentiation. Because this lncRNA overlaps with miR-221 in the genome, it was named miR-221 host gene (MIR221HG). The purpose of this study was to clone the full length of MIR221HG, detect its subcellular localization, and determine the effects of MIR221HG on bovine adipocyte differentiation. The 5′ rapid amplification of cDNA ends (RACE) and 3′ RACE analyses demonstrated that MIR221HG is a transcript of 1064 nucleotides, is located on the bovine X chromosome, and contains a single exon. Bioinformatics analyses suggested that MIR221HG is an lncRNA and the promoter of MIR221HG includes the binding consensus sequences of the forkhead box C1 (FOXC1) and krüppel-like factor5 (KLF5). The semi-quantitative PCR and quantitative real-time PCR (qRT-PCR) of nuclear and cytoplasmic fractions revealed that MIR221HG mainly resides in the nucleus. Inhibition of MIR221HG significantly increased adipocyte differentiation, as indicated by a dramatic increment in the number of mature adipocytes and in the expression of the respective adipogenic markers, peroxisome proliferator-activated receptor γ (PPARγ), CCAAT/enhancer-binding protein α (C/EBPα), and fatty acid binding protein 4 (FABP4). Our results provide a basis for elucidating the mechanism by which MIR221HG regulates adipocyte differentiation.

## 1. Introduction

Adipose tissue is an important endocrine organ with an extremely complicated role in maintaining body energy balance [1,2]. For livestock, fat is a crucial factor affecting economic traits, including growth, development, and meat quality. Specifically, intramuscular fat content is closely related to meat tenderness and flavor, and is an important indicator of meat quality [3,4]. Currently, animal breeding is moving towards an era of molecular breeding. The keys to molecular breeding are to clarify the biological and physiological bases of economically important traits and to elucidate the molecular mechanisms by which genes regulate such traits.

Adipogenesis is a complicated but precisely orchestrated process involving a series of transcription factors. Numerous studies have shown that the signal transduction network based on the regulatory factors, peroxisome proliferator-activated receptor γ (PPARγ) and CCAAT/enhancer-binding protein α (C/EBPα), plays important roles in adipogenesis [5,6,7]. However, many regulatory factors that participate in this biological pathway remain to be identified, and numerous details regarding the regulatory mechanism of adipogenesis need to be elucidated [8,9].

Long noncoding RNAs (lncRNAs) are a class of RNA transcripts longer than 200 nt that lack the protein coding ability and can contain or lack a polyadenosine (polyA) tail [10]. Transcripts produced by approximately 4% to 9% of the mammalian genome are lncRNAs [11], which were originally considered “junk” genomic sequences with no biological function [12]. In recent years, researches from the Functional Annotation of the Mammalian Genome (FANTOM) and Encyclopedia of DNA Elements (ENCODE) projects have shown that lncRNAs play crucial roles in multiple biological processes, including chromatin modification, cell cycle regulation, genomic imprinting, cell differentiation, and X chromosome silencing [13,14,15,16].

Studies have also shown that lncRNAs have important regulatory effects on adipogenesis [17,18,19]. Steroid receptor RNA activator (SRA) was the first identified lncRNA with a regulatory effect on adipogenesis. SRA expression increased approximately 2-fold during 3T3-L1 preadipocytes differentiation and it promoted 3T3-L1 cells differentiation by enhancing the transcriptional activity of PPARγ [17]. Microarray analysis revealed that SRA is involved in multiple biological processes in adipocytes, including the cell cycle and insulin-related signal transduction, suggesting that SRA may promote adipogenesis through a variety of pathways [17]. miR-140 knockout in adipocyte-derived stem cells (ADSCs) decreased the expression of a nuclear lncRNA nuclear paraspeckle assembly transcript 1 (NEAT1) and weakened the potential for adipogenesis. RNA pulldown and fluorescence in situ hybridization experiments indicated that miR-140 binds to NEAT1 in the nucleus. miR-140 overexpression increased NEAT1 stability and expression and promoted the expression of genes controlling lipid droplet formation and adipocyte differentiation, such as PPARγ and C/EBPα [18]. The adipocyte differentiation-associated long noncoding RNA (ADNCR) was mainly localized in the cytoplasm of preadipocytes and bound to miR-204, which increased the expression of the miR-204 target gene, *silent information regulator 1 (SIRT1)*, and then inhibited adipocyte differentiation [19].

Thus far, there are few reports on lncRNAs in cattle; the only relevant studies have reported the discovery and characterization of lncRNAs. Huang et al. employed existing expressed sequence tags to reassemble transcripts and identified 449 lncRNAs in intergenic regions [20]. Characteristics analysis indicated that these lncRNAs have significant tissue specificity and are moderately conserved in mammals. Lambros et al. used RNA-Seq data from 18 bovine tissue samples to identify and annotate 9778 new lncRNAs, including the metastasis-associated lung adenocarcinoma transcript 1 (MALAT1) and HOX transcript antisense RNA (HOTAIR), which are well-known lncRNAs in humans and mice [21]. Other lncRNAs reported in cattle include MEG8, MEG8-IT1, MEG8-IT2, MEG8-IT3, and LINC24061 [22,23]. These studies enrich the annotation of the cattle genome and provide valuable resources for the subsequent functional identification of lncRNAs.

In our previous study, we identified a novel lncRNA that was differentially expressed during bovine adipocyte differentiation [19]. Because this lncRNA overlaps with miR-221 in the genome, it was named miR-221 host gene (MIR221HG). The purpose of this study was to clone the full length of MIR221HG, detect its subcellular localization, and determine the effects of MIR221HG on bovine adipocyte differentiation.

## 2. Materials and Methods

### 2.1. Sample Collection

All animal experiments were carried out in accordance with the guidelines of the Institutional Administrative Committee and Ethics Committee of Laboratory Animals (license number: SYXK [Su] 2017-0044) and were approved by the Yangzhou University Institutional Animal Care and Use Committee.

Subcutaneous adipose tissue was collected from 24-month-old Simmental cattle at a slaughterhouse in Nanjing, Jiangsu Province, China. The samples were collected immediately after slaughter using strict aseptic procedures to avoid contamination, placed in high-glucose Dulbecco’s modified eagle medium (DMEM) containing 10% fetal bovine serum (FBS) and 1% penicillin-streptomycin (P/S), and then brought back to the laboratory as soon as possible for bovine adipocyte-derived stem cells (bADSCs) culture.

### 2.2. Cell Culture

The bADSCs were cultured according to our previously reported method [24]. The potentially contaminated epidermis, blood vessels, and connective tissue were carefully removed under sterile conditions, and the remaining tissue was washed 3 times in phosphate-buffered saline (PBS) containing 1% penicillin/streptomycin and cut into approximately 1-mm^3^ pieces. The tissue pieces were distributed evenly in a flask using a 1-mL pipette. The flask was gently flipped so that the side with the tissue pieces was facing up. An appropriate amount of high-glucose DMEM containing 20% FBS and 1% penicillin/streptomycin was added, and the flask was capped and placed in a 37 °C incubator containing 5% CO_2_. Six hours later, after the tissue pieces had attached to the cell culture flask, the flask was carefully turned over and incubated in the typical position. After 10 days, the tissue pieces were removed, and the attached cells were washed twice with PBS, trypsinized, centrifuged at 250× *g* for 10 min, and passaged or frozen according to the experimental needs.

### 2.3. Adipocytes Differentiation and Oil Red O Staining

Adipocytes differentiation: When the cells reached 100% confluence, the medium was replaced with a differentiation-inducing medium (complete medium containing 10 μg/mL insulin, 1 μM dexamethasone, 0.5 mM 3-isobutyl-1-methylxanthine (IBMX), and 1 μM rosiglitazone, all purchased from Sigma). After 3 days of induction, the differentiation-inducing medium was carefully discarded, and differentiation-maintaining medium (complete medium containing 10 μg/mL insulin and 1 μM rosiglitazone) was added and changed every 2 days. After 10 days, the cells were harvested for quantitative real-time PCR (qRT-PCR) or Western blotting analysis.

Oil red O staining: After adipocyte differentiation, the medium was discarded, and the cells were washed with PBS and fixed in 10% formalin for 5 min. The 10% formalin was discarded, the same volume of formalin was added, and the cells were incubated for 1 h. The formalin was discarded, and the cells were washed with 60% isopropanol. The flask was completely dried, and oil red O working solution (0.3% oil red O, 60% isopropanol, and 40% PBS) was added for 20 min at room temperature. The oil red O solution was discarded, and the cells were washed immediately 4 times with PBS. Pictures were taken under a microscope.

### 2.4. RNA Extraction, cDNA Synthesis, and qRT-PCR

Total RNA was extracted from the cells using RNAiso Plus (TaKaRa, Dalian, China). The RNA quality was measured using a NanoDrop 1000 spectrophotometer (Thermo Scientific, Wilmington, DE, USA). Reverse transcription was performed using a PrimeScript^TM^ RT reagent kit with gDNA Eraser (TaKaRa) according to the manufacturer’s instructions. qRT-PCR was conducted on a Bio-Rad CFX96 real-time PCR instrument in a reaction volume of 25 μL (12.5 μL of SYBR Premix Ex Taq II, 10 μM forward and reverse primers, and 10 ng of cDNA) with the following reaction program: 95 °C for 30 s, followed by 40 cycles of 95 °C for 5 s and 60 °C for 30 s. Glyceraldehyde-3-phosphate dehydrogenase (*GAPDH*) and hypoxanthine phosphoribosyltransferase 1 (*HPRT1*) were used as internal controls, and gene expression levels were calculated using the 2^−∆∆CT^ method [25]. The qRT-PCR primers were designed using Beacon Designer 8.12, and the primer sequences are listed in Table S1.

### 2.5. 5′- and 3′-Rapid Amplification of cDNA Ends (RACE)

The 5′ and 3′ ends of MIR221HG were obtained by the RACE assay using a SMARTer RACE cDNA Amplification Kit (Clontech, Palo Alto, CA, USA) according to the manufacturer’s protocol. Total RNAs extracted from bADSCs and differentiated adipocytes were used. The specific 5′ and 3′ primer sequences for the RACE assay were CACTGGGCTTAATCATTGGACAGAGTGC and GAAGGGTAGCATTACACCTGGTCTCTGG, respectively.

### 2.6. Subcellular Localization of MIR221HG

Cytoplasm and nuclear RNA was isolated from bADSCs and differentiated adipocytes using a PARIS kit (Life Technologies, Carlsbad, CA, USA) according to the instructions. Semi-quantitative PCR and qRT-PCR were used to detect MIR221HG expression. For semi-quantitative PCR, the reaction specificity was enhanced by using touchdown PCR with the following reaction program: Predenaturation at 94 °C for 4 min; 18 cycles of denaturation at 94 °C for 30 s, annealing at 68 °C for 30 s, and extension at 72 °C for 30 s, with a 1 °C decrease in the annealing temperature in each cycle; 10 cycles of denaturation at 72 °C for 30 s, annealing at 50 °C for 30 s, and extension at 72 °C for 30 s; and final extension at 72 °C for 10 min. The primers are listed in Table S1.

### 2.7. RNA Interference (RNAi)

The short interfering RNAs (siRNAs) specifically targeted to MIR221HG and negative control siRNA (NC siRNA) were purchased from GenePharma (Shanghai, China). The siRNA sequences for MIR221HG were MIR221HG-si1, 5′ UCAAUAGAAUUGCCUGCU 3′ and MIR221HG-si2, 5′ UAAGCCCAGUGGUUUAUGC 3′. To detect the interference efficiencies of MIR221HG-specific siRNAs, the siRNAs were transfected into preadipocytes. Forty-eight hours after transfection, total RNA was extracted and the expression levels of MIR221HG were measured by qRT-PCR. To explore the effects of MIR221HG on bovine adipogenesis, bADSCs were transfected with MIR221HG-si1 and induced to differentiate. After 10 days, oil red O staining and qRT-PCR analysis were performed. 

### 2.8. Western Blotting

The cells were washed twice with PBS, and 200 μL of high-efficiency radio-immunoprecipitation assay (RIPA) lysis buffer containing phenylmethanesulfonyl fluoride (PMSF, Solarbio, Beijing, China) was added to each well of a 6-well plate. After cell lysis, the cell lysates were incubated at 100 °C for 5 min in a PCR machine for protein denaturation and then centrifuged at 12,000× *g* for 10 min. Equal volumes of supernatant were separated by 10% sodium dodecyl sulfate-polyacrylamide gel electrophoresis (SDS-PAGE). Proteins were transferred to a polyvinylidene difluoride (PVDF) membrane (Amersham Biosciences, Little Chalfont Buckinghamshire, UK) using a semidry method. The membrane was blocked with 5% skim milk for 2 h, incubated with primary antibody overnight at 4 °C, washed 3 times for 10 min each with tris-buffered saline and Tween 20 (TBST) at room temperature on a destain shaker, incubated with secondary antibody for 2 h at room temperature, washed 3 times for 10 min each with TBST, neutralized with deionized water for 5 min, subjected to color development with ECL Plus, and imaged using a ChemiDoc XRS+ system (Bio-Rad, Hercules, CA, USA). PPARγ (catalog #ab45036), C/EBPα (catalog #ab140479), and fatty acid binding protein 4 (FABP4, catalog #ab92501) antibodies were purchased from Abcam (Cambridge, UK), and the GAPDH antibody (catalog #sc-47724) was purchased from Santa Cruz Biotechnology (Santa Cruz, CA, USA).

### 2.9. Statistical Analysis

Data processing and Student’s *t* test were performed using GraphPad Prism 8. Data are presented as the mean ± standard error (SE). *p* < 0.05 indicates a significant difference.

## 3. Results

### 3.1. lncRNA MIR221HG Downregulated during Bovine Adipocyte Differentiation

**Figure 1:**
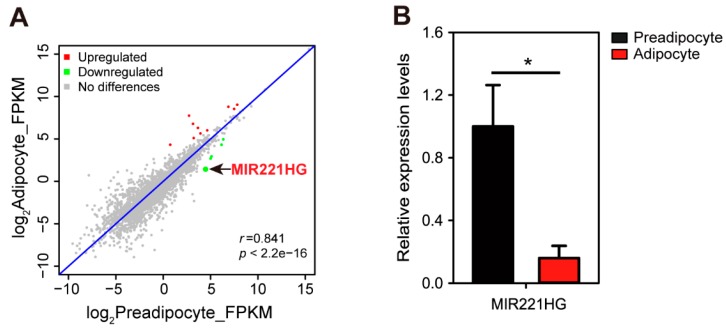
long noncoding RNA miR-221 host gene (MIR221HG) is downregulated in adipocyte differentiation. (**A**) A scatter plot shows the correlation of lncRNA abundance between preadipocytes and adipocytes. The red dots represent the upregulated lncRNAs in adipocytes; the green dots represent the downregulated lncRNAs. (**B**) Validation of lncRNA MIR221HG. Total RNA of preadipocytes and adipocytes were respectively extracted, and qRT-PCR was conducted to verify the differential expression of MIR221HG. The data are presented as mean ± SE from three independent experiments. * *p* < 0.05.

To identify lncRNAs involved the bovine adipocyte differentiation and function, we analyzed the differentially expressed lncRNAs in bovine preadipocytes (bADSCs) and mature adipocytes. This analysis identified a novel lncRNA, which was robustly downregulated in the process of bovine adipocyte differentiation and overlapped with miR-221 in the genome (Figure 1A). Therefore, we named it miR-221 host gene (MIR221HG). The qRT-PCR analysis confirmed that MIR221HG expression was downregulated by approximately 75% during bovine adipocyte differentiation (Figure 1B).

### 3.2. Characterization of the MIR221HG Sequence

**Figure 2:**
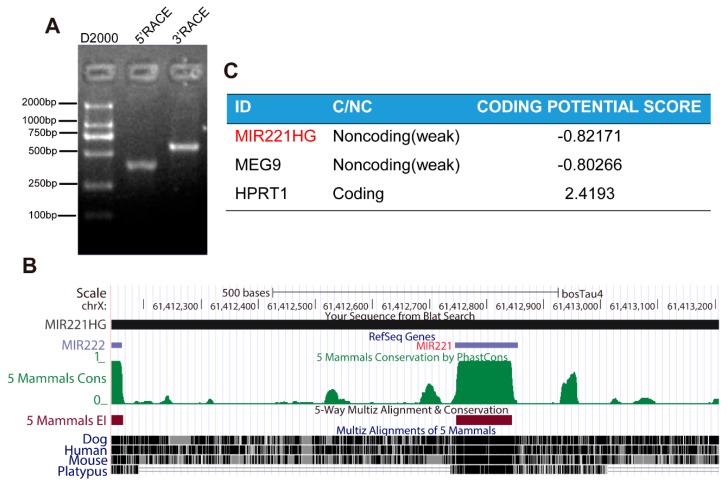
(**A**) The 5′ rapid amplification of cDNA ends (RACE) and 3′ RACE analyses illustrated that MIR221HG is a transcript of 1640 nucleotides with one single exon. (**B**) Genome structures of MIR221HG. Sequence conservation of MIR221HG was measured by multiple alignments of five vertebrate species using the UCSC Genome Browser. (**C**) The Coding Potential Calculator (CPC) program was used to evaluate the coding potential of MIR221HG, maternally expressed gene 9 (MEG9), and hypoxanthine phosphoribosyltransferase 1 (HPRT1), and both MIR221HG and MEG9 were predicted to be non-coding RNAs, while HPRT1 was identified to code for protein.

RACE is a common method for cloning full-length lncRNAs; with this convenient, fast, and efficient method, one can rapidly amplify the 5′ and 3′ ends of low copy number transcripts [26]. Therefore, a SMARTer RACE cDNA Amplification Kit was used to amplify the 5′ and 3′ ends of the lncRNA MIR221HG. This analysis showed that MIR221HG is 1064 nucleotides in length, is located on the bovine X chromosome, and contains a single exon (Figure 2A,B). The full sequence of MIR221HG is presented in Figure S1. Alignments using the UCSC genome browser showed that MIR221HG and miR-221 overlap in the genome and that the MIR221HG sequence is poorly conserved among species (Figure 2B).

To prove that MIR221HG is a true lncRNA, the coding potential of MIR221HG, MEG9 (a known lncRNA) and HPRT1 (a known protein-coding gene) were predicted using the online tool Coding Potential Calculator [27]. MIR221HG was predicted to have very low coding potential, with a score of approximately −0.8, similar to MEG9, a well-known lncRNA (Figure 2C), while the score for HPRT1 was much higher at 2.4193 (Figure 2C). These results strongly suggested that MIR221HG is an lncRNA.

### 3.3. Multiple FOXC1 and KLF5 Binding Sites Are Present in the MIR221HG Promoter Region

**Figure 3:**
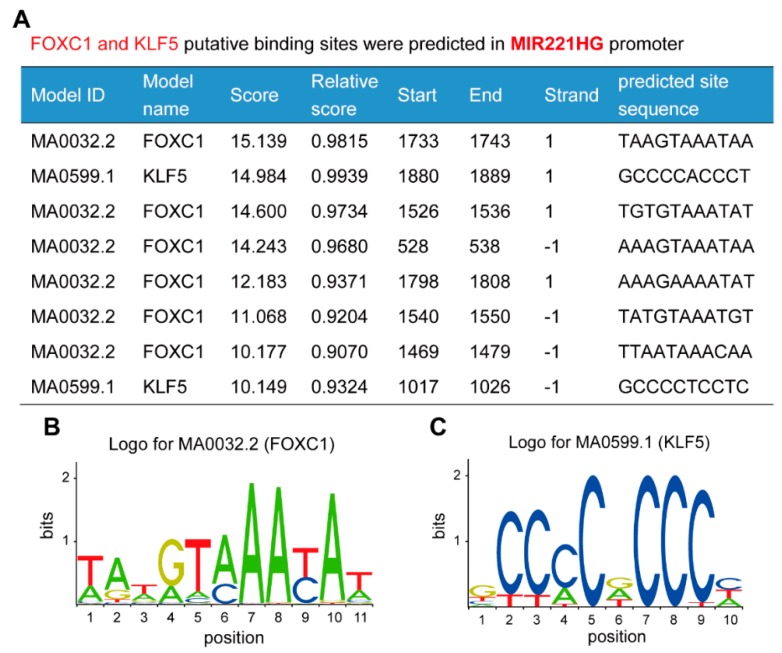
Multiple forkhead box C1 (FOXC1) and krüppel-like factor5 (KLF5) putative binding sites are predicted in the MIR221HG promoter region. (**A**) Predicted FOXC1 and KLF5 binding sites within a 2000-bp region of the MIR221HG promoter. (**B**) FOXC1 binding motif sequence logo from JASPAR. (**C**) KLF5 binding motif sequence logo from JASPAR.

Gene expression is regulated by a complex network of transcription factors. Analysis using the online database JASPAR showed that the MIR221HG promoter region contains binding sites for many adipocyte differentiation-related transcription factors, such as forkhead box C1 (FOXC1), Kruppel-like factor 5 (KLF5), specificity protein 1 (SP1), signal transducer and activator of transcription 1 (STAT1), and C/EBPα. Notably, there are six FOXC1 binding sites and two KLF5 binding sites within a 2000-bp region of the MIR221HG promoter (Figure 3), and the sequences of these bindings sites are highly conserved, suggesting that MIR221HG may be tightly regulated by FOXC1 and KLF5.

### 3.4. MIR221HG Mainly Resides in the Nucleus

**Figure 4:**
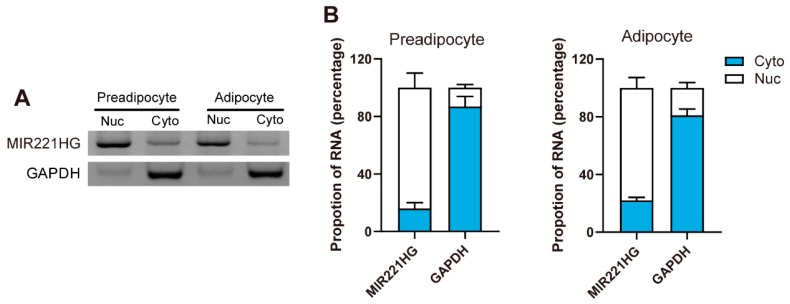
MIR221HG is a nucleus lncRNA. (**A**,**B**) MIR221HG in cytoplasmic (Cyto) and nuclear (Nuc) fractions of preadipocytes and adipocytes was determined by semi-quantitative PCR (**A**) and qRT-PCR (**B**). Glyceraldehyde-3-phosphate dehydrogenase (GAPDH) was used as markers for the cytoplasmic fraction. The data are representatives of three independent experiments.

lncRNAs have various modes of action, and different subcellular localization may indicate different regulatory mechanisms. Cell fractionation followed by semi-quantitative PCR demonstrated that MIR221HG mainly resides in the nucleus of preadipocytes and adipocytes (Figure 4A). This was also supported by qRT-PCR, which revealed that about 75% of the spliced MIR221HG transcript resides in the nucleus (Figure 4B). These results suggested that MIR221HG may function in the nucleus and act as a regulatory lncRNA involved in transcriptional control of adipogenic genes’ expression during adipocyte differentiation.

### 3.5. MIR221HG Inhibited Bovine Adipocyte Differentiation

**Figure 5:**
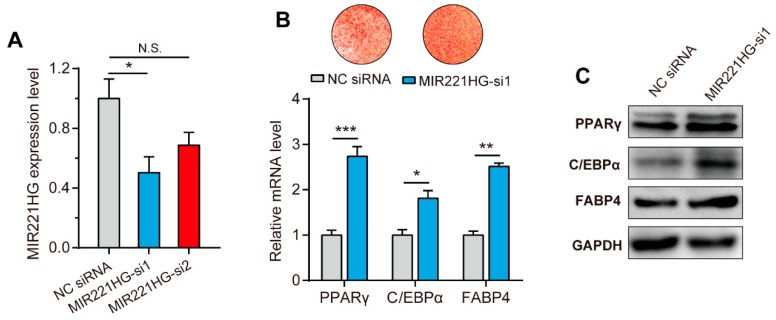
Inhibition of MIR221HG promotes bovine adipocyte differentiation. (**A**) Interference efficiencies of MIR221HG-specific short interfering RNAs (siRNAs). siRNAs were transfected into preadipocytes. Forty-eight hours after transfection, total RNA was isolated and the expression levels of MIR221HG were detected by qRT-PCR. (**B**,**C**) siRNA1-mediated downregulation of MIR221HG promotes the expression of adipogenic markers peroxisome proliferator-activated receptor γ (PPARγ), CCAAT/enhancer-binding protein α (C/EBPα), and fatty acid binding protein 4 (FABP4) at both mRNA (**B**) and protein (**C**) levels. The data are presented as mean ± SE from three independent experiments. * *p* < 0.05; ** *p* < 0.01; *** *p* < 0.001.

To explore the effects of MIR221HG on adipocyte differentiation, two MIR221HG short interfering RNAs (siRNAs) sequences were designed and transfected into bADSCs. Forty-eight hours after transfection, total RNA was extracted, and MIR221HG expression levels were determined by qRT-PCR, which showed that the siRNA1 significantly decreases MIR221HG expression by 55% (*p* < 0.05, Figure 5A). 

bADSCs were then transfected with MIR221HG siRNA1 and induced to differentiate. After 10 days, oil red O staining and qRT-PCR analysis were performed. Inhibition of MIR221HG expression resulted in a significant increase in the number of lipid droplets compared to control transfection (Figure 5B, upper panel) and the significant upregulation of the adipocyte differentiation marker genes, PPARγ, C/EBPα, and FABP4 (*p* < 0.01, Figure 5B, lower panel). Accordingly, increased protein expression of PPARγ, C/EBPα, and FABP4 were also detected (Figure 5C). These results indicated that inhibition of MIR221HG promotes bovine adipocyte differentiation.

## 4. Discussion

In recent years, with the development of emerging biotechniques and a deeper understanding of the genome, researchers have found that approximately 70% of genomic sequences are transcribed and that a large number of lncRNAs are produced [28,29]. However, due to their low expression level and lack of sequence conservation, lncRNAs have long been ignored by researchers [28,30]. The ENCODE project revealed that 80% of the human transcriptome is functional and that sequences previously identified as “junk” play important roles in controlling the functions of cells, tissues, and organs. Currently, tens of thousands of lncRNAs have been identified in different species using high-throughput sequencing techniques [31,32,33,34,35].

Cattle are an important economic animal. Studies on the regulation of fat development in cattle have mainly focused on gene structure analysis and on the investigation and functional analysis of key gene expression profiles [36,37]. There have been few studies on the regulation of bovine adipocyte differentiation by lncRNAs, and many regulatory mechanisms are still unclear. In this study, the full-length sequence of the lncRNA MIR221HG was cloned for the first time using RACE. MIR221HG is a transcript with 1064 nucleotides and is located on the bovine X chromosome. The subcellular localization assay showed that MIR221HG mainly localized in the nucleus of preadipocytes and adipocytes. Silencing MIR221HG in bADSCs resulted in an increase in the number of fat droplets and significantly upregulated the expression of the adipocyte differentiation markers, PPARγ, C/EBPα, and FABP4, suggesting that MIR221HG has an important regulatory effect on bovine adipocyte differentiation.

Alignments using the UCSC genome browser revealed the overlapped genomic positions of MIR221HG and miR-221. The expression of miR-221 was significantly downregulated during adipocyte differentiation, and overexpression of miR-221 significantly suppressed adipogenesis [38]. In human adipose-derived stem cells, elevated tumor necrosis factor-α (TNFα) mRNA levels resulted in decreased miR-221 expression. Moreover, miR-221 expression in adipose tissue was negatively correlated with body mass index (BMI) [39]. These results suggested that miR-221 has an inhibitory effect on adipocyte differentiation. As MIR221HG and miR-221 overlapped in the genome, MIR221HG may inhibit adipocyte differentiation through regulating miR-221 expression. However, the regulatory relationship between MIR221HG and miR-221 and their effects on adipocyte differentiation still remains to be further investigated.

The regulatory mechanisms of action of lncRNAs are complicated. Most lncRNAs are located in the nucleus, where they act as molecular scaffolds, assist in alternative splicing, and modify chromosome conformations [40,41,42]. However, some lncRNAs, such as long intergenic noncoding RNA-muscle differentiation 1 (linc-MD1), imprinted maternally expressed transcript (H19), and long intergenic non-coding RNA for kinase activation (LINK-A), promote or inhibit translation or competitively adsorb miRNAs in the cytoplasm [16,43,44]. MIR221HG is mainly expressed in the nucleus of preadipocytes, indicating that it may regulate gene expression at the transcriptional level.

The MIR221HG sequence is poorly conserved among different species. The lack of primary sequence conservation in lncRNAs is a hot topic in the scientific community. Some researchers contend that this low conservation is contrary to the function [45]. However, accumulating studies have shown that lncRNAs with poor sequence conservation may show functional conservation. For example, Gong et al. found that LncMyoD regulates mouse skeletal muscle differentiation by repressing insulin-like growth factor 2 mRNA binding protein 2 (IMP2)-mediated mRNA translation; however, no human homologue of LncMyoD was identified by sequence alignment [46]. Further analysis identified lncRNA-hLncMyoD at a similar position in the human genome, and conserved MyoD binding sites were present in the promoters of hLncMyoD and mouse LncMyoD. The overexpression of hLncMyoD in LncMyoD-knockout mouse myofibroblasts rescued impaired muscle formation, suggesting that LncMyoD is functionally conserved. Although LncMyoD and hLncMyoD were conserved regarding genome location and function, their sequence conservation is extremely low [46]. lncRNAs and mRNAs are under different selective pressures during evolution, and sequence conservation cannot be used as a simple standard to measure the importance of lncRNA function. In fact, the primary RNA sequences of X-inactive specific transcript (XIST) and telomerase RNA component (TERC), the most thoroughly studied mammalian lncRNAs, are not conserved in humans and mice, but they perform the same function in both species [47].

## 5. Conclusions

In conclusion, we identified a novel lncRNA, MIR221HG, located on the X chromosome with a full length of 1064 nucleotides. It was mainly expressed in the cytoplasm and had an inhibitory effect on adipocyte differentiation. The results of this study provide a basis for elucidating the mechanism by which MIR221HG regulates adipocyte differentiation.

## Supplementary Materials

The following are available online at https://www.mdpi.com/2073-4425/11/1/29/s1, Figure S1: Nucleotide sequence of lncRNA MIR221HG, Table S1: Specific sequence used for semi-quantitative PCR and qRT-PCR.
