# Supplementary material for: MIR221HG Is a Novel Long Noncoding RNA that Inhibits Bovine Adipocyte Differentiation
Source: Genes (Basel). 2019 Dec 26;11(1):29. doi: 10.3390/genes11010029 (PMC7016960; doi:10.3390/genes11010029)
Supplement: Supplementary file 1 [file genes-11-00029-s001.zip › Table S1.docx]

**Table S1**. Specific sequence used for semi-quantitative PCR and qRT-PCR.

| Sequence Definition | Product Length(bp) | Sense Primer | Antisense Primer | Use |
| --- | --- | --- | --- | --- |
| MIR221HG | 371 | CCTGAGTGAGATTATTGAGAAG | AATGGATGGATGGATGGATG | semi-quantitative PCR |
| GAPDH | 365 | AAGTTCAACGGCACAGTCA | GTCATAAGTCCCTCCACGAT | semi-quantitative PCR |
| PPARγ | 121 | AGGATGGGGTCCTCATATCC | GCGTTGAACTTCACAGCAAA | qRT-PCR |
| C/EBPα | 130 | TGGACAAGAACAGCAACGAG | TTGTCACTGGTCAGCTCCAG | qRT-PCR |
| FABP4 | 111 | AAGTCAAGAGCATCGTAA | CCAGCACCATCTTATCAT | qRT-PCR |
| MIR221HG | 75 | ATGCTTCACCTATACTGTT | CACTCTGTCCAATGATTAAG | qRT-PCR |
| GAPDH | 91 | CACTCACTCTTCTACCTT | GCCAAATTCATTGTCGTA | qRT-PCR |
| HPRT1 | 91 | AAGCCTAAGATGAGAGTC | CCACAGAACAAGAACATT | qRT-PCR |
