# Supplementary figures and images for: MIR221HG Is a Novel Long Noncoding RNA that Inhibits Bovine Adipocyte Differentiation
Source: Genes (Basel). 2019 Dec 26;11(1):29. doi: 10.3390/genes11010029 (PMC7016960; doi:10.3390/genes11010029)

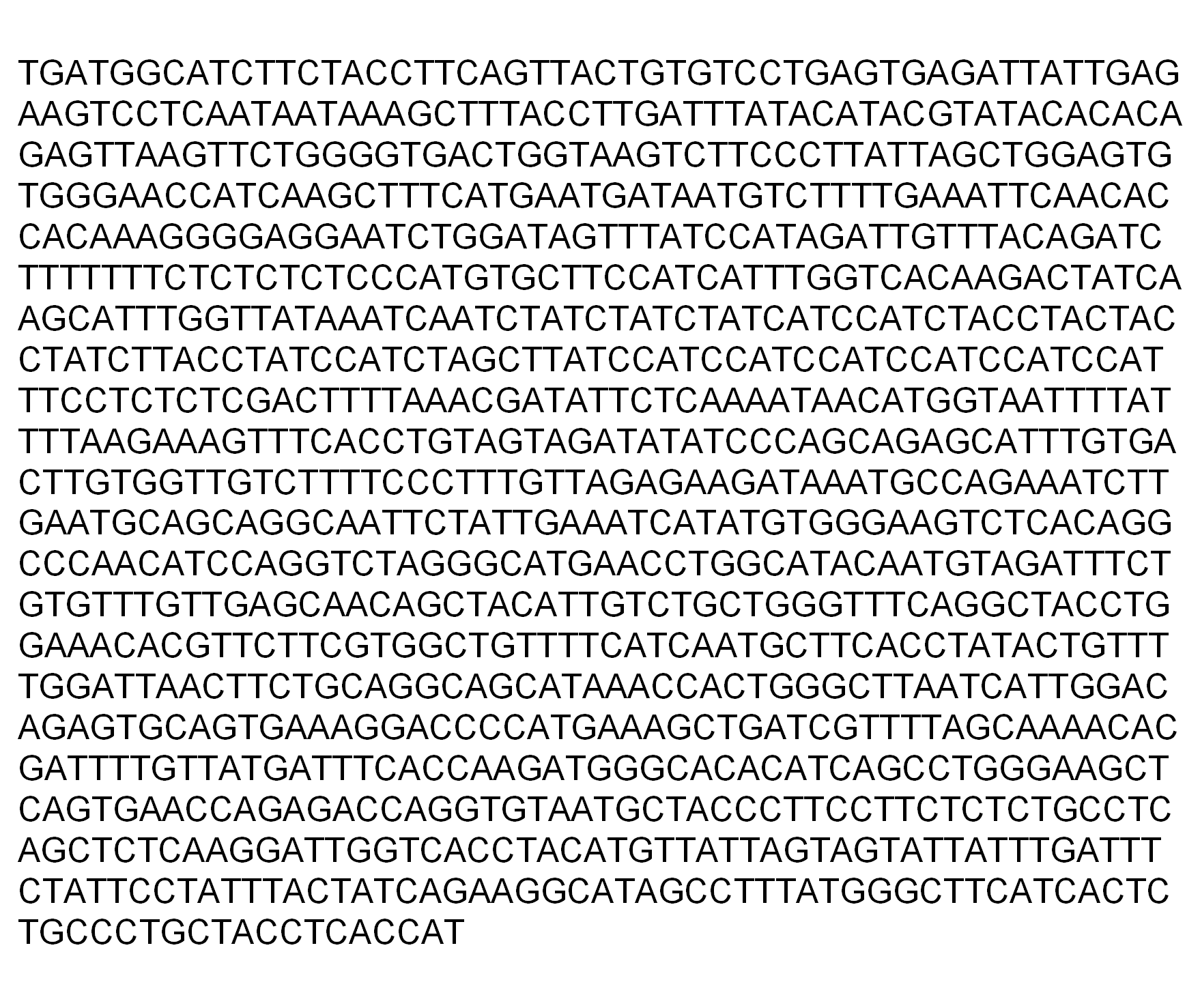

Supplement: Supplementary file 1 [file genes-11-00029-s001.zip › Figure S1 MIR221HG sequence.tif]
